# The Mediterranean archive of isotopic data, a dataset to explore lifeways from the Neolithic to the Iron Age

**DOI:** 10.1038/s41597-023-02783-y

**Published:** 2023-12-20

**Authors:** Martina Farese, Silvia Soncin, John Robb, Ricardo Fernandes, Mary Anne Tafuri

**Affiliations:** 1 Department of Environmental Biology and Mediterranean bioArchaeological Research Advances (MAReA) Centre, Sapienza University of Rome, Rome, Italy; 2 Department of Archaeology, University of Cambridge, Cambridge, UK; 3 Department of Archaeology, Max Planck Institute of Geoanthropology, Jena, Germany; 4 Department of Bioarchaeology, Faculty of Archaeology, University of Warsaw, Warsaw, Poland; 5 Climate Change and History Research Initiative, Princeton University, Princeton, USA; 6 Department of Archaeology and Museology, Faculty of Arts, Masaryk University, Brno, Czechia

**Keywords:** Biogeochemistry, Biochemistry

## Abstract

We present the open-access Mediterranean Archive of Isotopic dAta (MAIA) dataset, which includes over 48,000 isotopic measurements from prehistoric human, animal and plant samples from archaeological sites in the Mediterranean basin dating from the Neolithic to the Iron Age (ca. 6000 – 600 BCE). MAIA collates isotopic measurements (*δ*^13^C, *δ*^15^N, *δ*^34^S, *δ*^18^O and ^87^Sr/^86^Sr) alongside supporting information (e.g. chronology, location and bibliographic reference). MAIA can be used to explore past human and animal diets and mobility, reconstruct paleo-ecological and -climatic phenomena and investigate human-environment interaction throughout later prehistory in the Mediterranean. MAIA has multiple research applications and here we show how it can be used to evaluate sample preservation and identify data gaps to be addressed in future research. MAIA is available in an open-access format and can be employed in archaeological, anthropological, and paleo-ecological research.

## Background & Summary

The Mediterranean Archive of Isotopic dAta (MAIA) is a large-scale open-access dataset of isotopic measurements collected from published studies on the Mediterranean area, covering a temporal range from the beginning of the Neolithic to the end of the Iron Age (ca. 6000 to 600 BCE).

Isotopic analyses have been used for the last forty years to reconstruct dietary practices and mobility patterns of archaeological and modern material^1–5^. Stable isotope ratios of carbon (*δ*^13^C), nitrogen (*δ*^15^N) and sulphur (*δ*^34^S) measured in bone and tooth collagen are primarily employed for dietary reconstructions. In humans, bones typically average diets for a number of years prior to death. The time span that these reflect depends on bone-specific collagen remodelling rates^6^, while in contrast, teeth do not undergo remodelling following collagen formation and can therefore be used to study diets from childhood to early adulthood^7^. Measurement of carbon and oxygen stable isotopes (*δ*^18^O) from bone bioapatite and tooth enamel offers dietary and spatial mobility information, respectively. Given that oxygen isotopes in consumers are sourced from ingested water, they can also be used in paleoclimatic studies and standard materials for such type of research include mollusc shells^8–10^. Another common isotopic proxy in archaeological research is strontium isotopes (^87^Sr/^86^Sr), typically measured in tooth enamel, mollusc shells and plant materials, used for spatial mobility studies^11^.

The proper interpretation of isotopic data often requires the use of ancillary data. For instance, when reconstructing human mobility and diets, it is necessary to compare human isotopic values with appropriate isotopic baselines established using local and coeval animal, plant, and soil samples^12,13^.

**Fig. 1:**
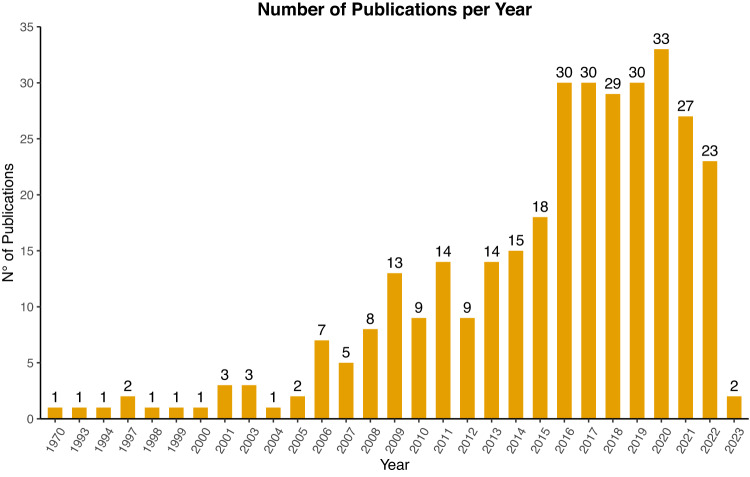
Number of publications on Mediterranean prehistoric sites included in the dataset divided by year. Total number of publications = 333.

Numerous studies of stable isotopes applied to archaeological materials have been published since the pioneering work by Vogel and Van Der Merwe on early maize cultivation^1^. However, most of these focus on limited archaeological contexts or, most commonly, on a single site. During the last 30 years, isotope analyses have been extensively applied in the Mediterranean region to reconstruct, among others, prehistoric lifeways. Since 1993^14^, more than three hundred articles and doctoral theses have been published that employ isotopic data to discuss palaeoecological scenarios and human and animal behaviour (Fig. 1).

The application of isotope analysis to archaeological remains has been crucial in observing how past communities dealt with social and economic changes throughout time^15–19^. Later prehistory, in particular, incorporates several key transformations that influenced humans both on a local and a broader scale: from the rise of farming economies in the Neolithic and the development of metalworking in the Copper Age to the increasing social stratification of the Bronze Age and the local fragmentation of societies in the Iron Age. For instance, isotope analysis presented the first evidence of C_4_ plant consumption (specifically millets) in Europe during the Bronze Age^20^, even though millet was previously thought to have been consumed in Europe only from the Iron Age onwards^21,22^. This evidence provides information on how past communities dealt with the surrounding environment during the second millennium BCE^23^, and the growing literature on millet exploitation in prehistoric Europe is a testament to how isotope studies have often set new agendas. However, most of these studies remained focused on site-specific questions with chronologically and geographically limited insight. This calls for a more thorough, comprehensive perspective on the isoscape of Mediterranean prehistory, which would be key in elucidating such dynamics at a broader scale.

The time is ripe for creating an open-access dataset where researchers can find all the isotopic data and archaeological information needed for studies applied to Mediterranean prehistoric contexts: the MAIA dataset. Collecting chrono- and geo-referenced isotopic data in a public dataset enables the study of spatial and temporal isotopic variations in plant, animal and human groups on a large scale. Given the lack of literary sources in prehistory, extensive studies are essential to deepening our understanding of prehistory in the Mediterranean basin. Researchers will also benefit from the integration of isotopic data with archaeological and anthropological information for a multi-proxy approach. Archives such as MAIA could also be used to detect which regions and chronological phases lack data (i.e. data gaps) and therefore guide future research. Alternatively, they can be explored to evaluate diagenetic alterations of the archaeological material relative to its chronology or geography. Recently, several datasets containing isotopic data from different time periods and geographical locations have been published, and the relevance and potential of this approach have been proven^24–29^. Following, we provide examples to demonstrate its research potential.

### Data gaps

Data gaps consist of areas and periods underrepresented or uninvestigated in the scientific literature. In the field of isotopic studies, they can arise from local environmental conditions which are adverse to the preservation of bioarchaeological remains and the choice of burial practices. For instance, in arid environments skeletal remains are typically less well preserved^30–32^ while cremation can destroy all organic tissues^33^. Simultaneously, the intensity and extension of archaeological research vary across countries, depending on their archaeological background and research interests. Thanks to MAIA, it is now possible to report the state of isotopic pre-historical research in the Mediterranean basin. By identifying data gaps, we can single out areas and time periods that have been understudied and would benefit from new isotopic research programmes. Simultaneously, novel and less employed isotopic methods studies could contribute to resolving pending historical questions, including for sites on which some isotopic research has already been undertaken. This may include less commonly employed sulfur isotopic analysis, the extension of strontium measurements, or the use of compound specific isotopic analysis.

### Preservation issues

Bioarchaeological materials undergo diagenesis following deposition in soil^34–38^. These include physical, chemical and biological modifications^39^, and their rate depends on burial practices, burial duration, local environmental conditions and soil characteristics^34,35,39,40^. Arid environments are well-known for being adverse to the preservation of bone collagen^30,31,41^ and there are studies pointing to an association between the length of burial times and sample preservation^32,34^. Diagenetic trajectories will depend on the type of tissues and chemical and structural properties of bioarchaeological materials^42–44^. There are some methods employed to screen for tissue preservation. For example, in collagen, the most widely employed organic tissue for stable isotope analysis of carbon, nitrogen and sulphur, elemental criteria are used to evaluate contributions from foreign contaminants together with the yield of collagen (%) following its extraction from bone and teeth^45^. Standard elemental preservation proxies include carbon, nitrogen and sulphur content (%) and their atomic ratios (C:N, C:S, N:S). However, different authors have presented different ranges of acceptance for these criteria. DeNiro^39^ and Ambrose^46^ proposed that isotopic measurements are acceptable when the C/N atomic ratio is between 2.9 and 3.6; van Klinken^45^ also advises that collagen yields should be >0.5 wt%, %C around 35% and %N between 11 and 16%. As for sulphur isotope analysis, bone collagen preservation in mammals and birds is acceptable when 0.15% ≤ %S ≤ 0.35%, C/S ranges from 300 to 900 and N/S from 100 and 300. For fish instead: 0.40% ≤ %S ≤ 0.80%, C/S ranges from 125 to 225 and N/S from 40 to 80^47,48^. Similar criteria with wide acceptance within archaeological isotopic research are lacking for plant remains^49^, although some studies have started to introduce the percentage of the elements and the isotope ratio to identify diagenesis^50^.

## Methods

The collection of isotopic data started in February 2022 and ended in March 2023. Bibliographic research was carried out using the web search engine Google Scholar (https://scholar.google.com/) and extended by checking the bibliography of collected publications. English was used as the search language (keywords: e.g. “neolithic”,”copper age”, “bronze age”, “iron age”, “isotopes”, “human”, “animal”, “Croatia”, “Italy”) as this is the language most widely used in the field. However, articles written in other languages were occasionally included if referenced in English-written articles. We are aware that this represents a limitation, but this is also a natural constraint as the authors are not familiar with the other Mediterranean languages. Therefore, we urge researchers from the field and the area to implement our dataset with missing articles, following the main scope of the Pandora Initiative (https://pandoradata.earth/about). For the same reason, if needed, our colleagues can independently translate the dataset into other languages and upload it to the same repository, following CC-BY guidelines.

In addition, some articles had to be excluded, although they were pertinent to our scope. This was done because only a graphical analysis was provided, with no possibility to access the original data.

MAIA covers the Mediterranean region between the beginning of the Neolithic and the end of the Iron Age, ca. 6000 – 600 BCE. We defined the Mediterranean region as the ecozone of diffusion of the olive tree following Horden and Purcell^51^, which includes 22 modern countries: Albania, Algeria, Croatia, Cyprus, Egypt, France, Greece, Israel, Italy, Jordan, Lebanon, Libya, Malta, Morocco, Montenegro, Palestine, Portugal, Spain, Slovenia, Syria, Tunisia and Turkey.

Isotopic data have been collected for human, animal and plant samples for the time frame described. To widen the isotopic baseline, we included animal and plant materials used as reference material from earlier or later periods (from the Palaeolithic to modern samples). The dataset lists the following types of isotopic proxies:

- *δ*^13^C and *δ*^15^N and *δ*^34^S measured in collagen;
- *δ*^13^C and *δ*^18^O from both carbonate and phosphate fractions of tooth enamel, bone bioapatite, and mollusc shells;
- ^87^Sr/^86^Sr and ^84^Sr/^86^Sr from tooth and bone apatite and mollusc shells.

Most human and animal isotopic measurements are collected from skeletal remains and shells. However, a minor portion of the dataset derives from other bioarchaeological remains, such as antlers, hair, horns, intestines and plastrons.

Single compound measurements have not been included in the MAIA dataset, as no studies on Mediterranean prehistory are currently available.

Isotopic values were collected together with supporting information to describe the cultural and social contexts of the samples. This includes archaeological information (e.g. the cultural horizon ascribable to the archaeological site or the single individual analysed, the type of pottery found in the archaeological site or among the grave goods) and biological data (sex estimation, age at death, taxonomic classifications) as reported in the publications. When available, each individual’s archaeological and laboratory identifiers have also been included in the dataset (see the “Dataset Structure” file at the data repository).

Each sample entry also contains chronological information. A temporal range was given and preferably set using radiocarbon measurements when available. When radiocarbon dates were not available, the samples’ chronology was determined from material culture.

Every entry was also georeferenced using the latest World Geodetic System (WGS84) version with Latitude and Longitude coordinates in decimal degrees. The coordinates were retrieved from the original publications when available. Otherwise, Google Earth (https://earth.google.com/web/) was used to locate the archaeological site and extract its coordinates. For the latter, an uncertainty estimate (in km) was included “unc. Radius (km)” column to indicate the area within which the site was likely located.

## Data Records

MAIA (available in XLSX and CSV formats)^52^ has been deposited on the Pandora platform (https://pandoradata.earth/, data available since 2023) following FAIR and CARE principles. It contains data from 18 modern countries: Albania, Algeria, Croatia, Cyprus, France, Greece, Israel, Italy, Jordan, Lebanon, Libya, Malta, Morocco, Palestine, Portugal, Spain, Syria and Turkey. No isotopic data have been found in the Mediterranean region for Egypt, Montenegro, Slovenia and Tunisia for the prehistoric period.

MAIA is subdivided into three datasets according to sample type: animals, humans and plants. A total of 333 publications (papers, book chapters, Master’s and PhD theses) have been included in the dataset.

The Pandora platform allows data communities to self-manage their data and membership features, including assigning Digital Object Identifiers (DOIs) to deposit materials. It is also possible to link different datasets together, promoting research collaborations. For isotopic studies, in particular, the IsoMemo initiative (https://pandoradata.earth/group/isomemo-group) brings together several archaeological datasets to enable comparative studies across time and space.

MAIA is organised into different sections:

- Bibliographic data: each publication’s reference (in APA style) and its relative DOI have been reported. Given the collaborative nature of the dataset, fields are included for the citation of other datasets that could be used to retrieve isotopic data in the future.
- ID data: this section provides the unique identifiers of each entry (including radiocarbon lab codes).
- Biological data: the sex and age at death of humans (and a modest fraction of the animals). The ages reported in the publications have been converted into age groups following Buikstra and Ubelaker^53^.
- Taxonomic data: not present for humans. For animal and plant entries, this section contains the family, genus and species details and information on animals’ trophic levels and plant metabolism.
- Sampling data: the type of sampled material and the component used for isotopic analysis. This section also includes information on whether incremental analysis has been carried out.
- Site data: a section containing details on the site from which the samples originate, the modern country where the site is located, the type and age of the site, archaeological information (e.g. pottery, metallurgy), the site location (latitude and longitude information georeferenced using decimal coordinates relative to the WGS84 system).
- Chronological data: this section includes all the information available on the sample chronology, including radiocarbon dates. Relative dates have also been included based on material culture.
- Isotopic data: this section contains all the isotopic data, including the quality parameters and the laboratory used for mass-spectrometry measurements. When isotopic measurements were provided as population means, the number of samples is reported in this section.

For a more detailed explanation and examples, see the “Dataset Structure” file at the data repository.

## Technical Validation

For carbon, nitrogen and sulphur, when included in the original publications, the values for the criteria used to assess collagen quality (collagen yield, %C, %N, %S, C/N, C/S, N/S) are included in the dataset. Isotopic data that did not meet the quality criteria were equally included. Values currently considered unreliable can, in fact, be used for other purposes (i.e. to assess diagenesis, to understand better causal mechanisms that lead to contamination) and might be reconsidered in future methodological advances. Data falling outside the recommended parameters can be filtered out before data analysis.

Oxygen values are typically reported using the Vienna-Pee Dee Belemnite (V-PDB) or Vienna Standard Mean Ocean Water (V-SMOW). Simultaneously, oxygen isotopes are commonly measured on carbonate or phosphate ions^54^. In some archaeological publications, oxygen isotopic values measured in phosphates are reported as drinking water values^55,56^. Therefore, the dataset has separate fields to report oxygen isotopic values measured in phosphates or carbonates relative to V-PDB and V-SMOW, as well as drinking water values if reported in the publications (see the “Dataset Structure” file at the data repository).

Regarding strontium isotopes, several publications have highlighted the preservation of the endogenous isotopic signature in tooth enamel^57–61^. Bone material is instead more easily subject to contamination during the burial^59,62–66^. For this reason, the type of analysed material is easily identifiable in MAIA under the “Sampled Element” and “Analysed Component” fields (see the “Dataset Structure” file at the data repository).

## Usage Notes

The values collected in MAIA can be used for different research purposes, among which we envision:

1. Reconstruction of past human lifeways such as diet or spatial mobility;
2. Palaeoenvironmental and palaeoclimatic studies;
3. Reconstruction of agricultural practices such as manuring or irrigation;
4. Detection of research data gaps;
5. Assessment of sample preservation.

**Fig. 2:**
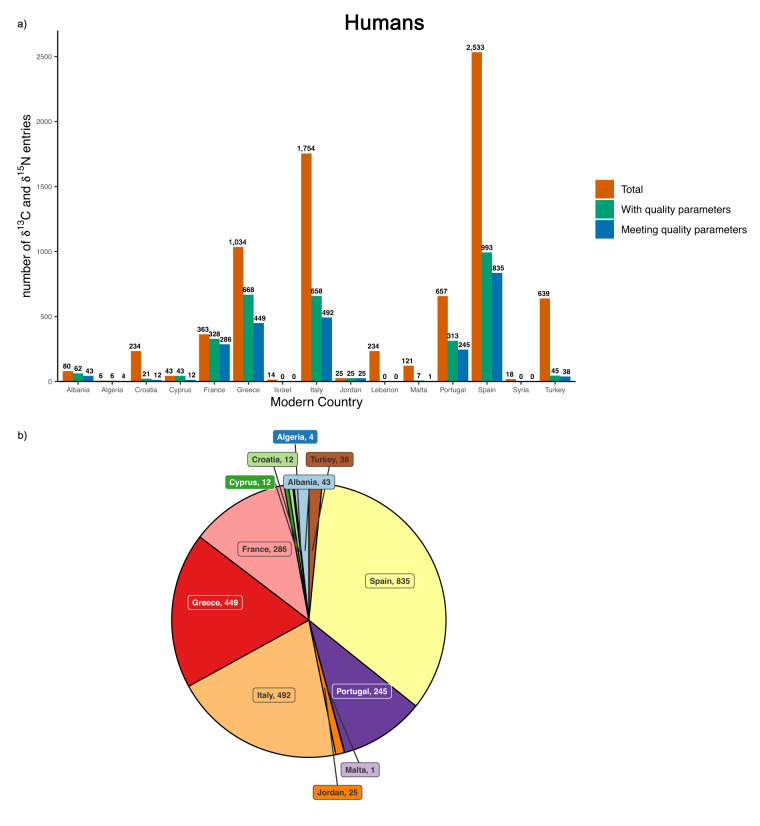
Human δ^13^C and δ^15^N preservation. (**a**) Bar chart showing in red the number of human samples with δ^13^C and δ^15^N measurements, in green the number of samples having all collagen quality criteria (collagen yield, %C, %N and C/N) reported and in blue the number of samples meeting the collagen criteria, divided by modern country. (**b**) Pie chart showing only the number of human samples with δ^13^C and δ^15^N measurements meeting the collagen quality criteria.

Here, we present an exploratory analysis of collagen preservation on prehistoric human and animal bones performed with MAIA, using the following collagen quality parameters for carbon and nitrogen measurements: collagen yield > 0.5 wt%, %C around 35%, %N between 11 and 16^45^ and C/N atomic ratio between 2.9 and 3.6^39^. A total of 7755 human samples with carbon and nitrogen measurements are available in MAIA. However, all the collagen quality criteria (collagen yield, %C, %N, C/N) are only available for 3169. As van Klinken^45^ suggested, combining all four parameters to evaluate collagen preservation is the best approach: 2442 (77.1%) samples meet all the quality criteria. Figure 2 shows the number of samples that meet the collagen quality criteria divided by modern country. Spain has the highest number of isotopic values, with 84.1% being good quality (835/993). The highest percentage of preservation can be found in Jordan. However, only 25 measurements are available for the country and therefore, the small sample size might not have captured the environmental effect on collagen preservation^30–32^.

**Fig. 3:**
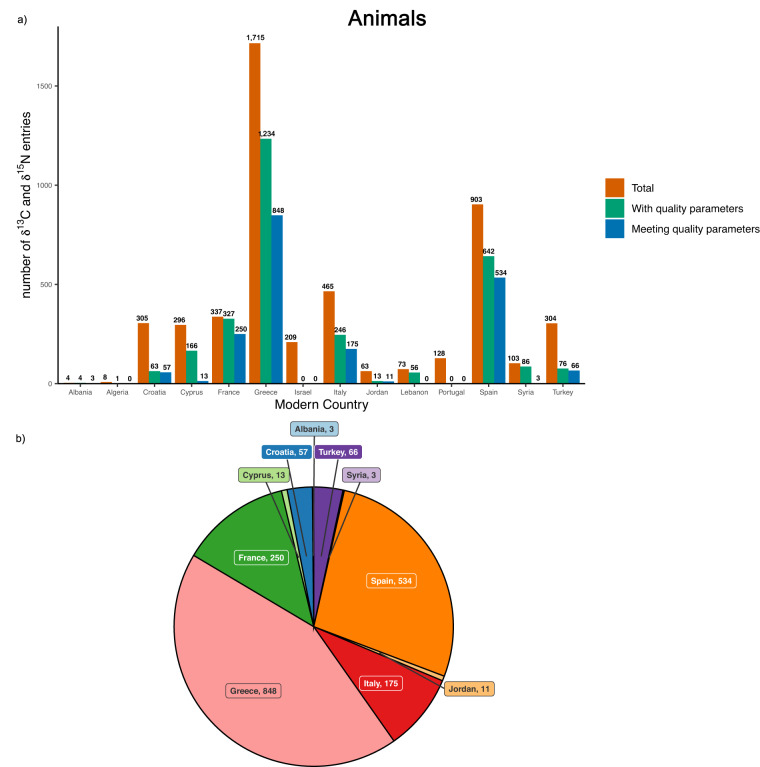
Animal δ^13^C and δ^15^N preservation. (**a**) Bar chart showing in red the number of animal samples with δ^13^C and δ^15^N measurements, in green the number of samples having all collagen quality criteria (collagen yield, %C, %N and C/N) reported and in blue the number of samples meeting the collagen criteria, divided by modern country. (**b**) Pie chart showing only the number of animal samples with δ^13^C and δ^15^N measurements meeting the collagen quality criteria.

Of the animal entries (4913) reporting carbon and nitrogen isotope values (with 36 entries from modern samples, therefore excluded from this preservation study), 2914 entries present all the collagen quality criteria (collagen yield, %C, %N, C/N) and 67.3% (1960 entries) of these meet the quality criteria. Greece shows the highest number of isotopic measurements within the quality ranges (848/1234). The number of entries meeting the quality criteria divided by modern country is reported in Fig. 3.

**Fig. 4:**
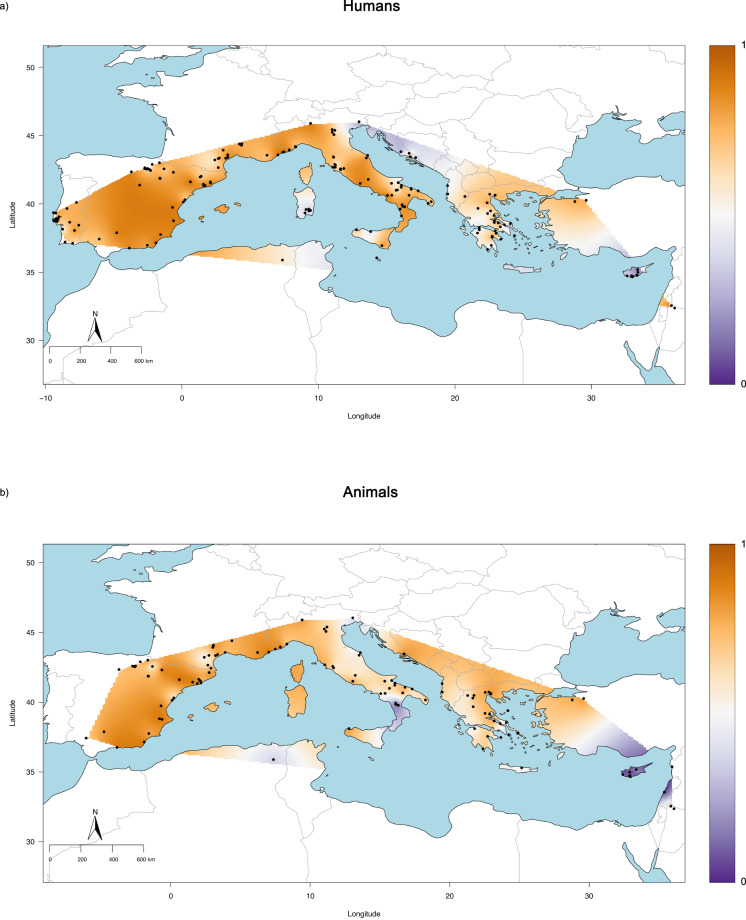
Distribution of preserved and non-preserved collagen for human and animal samples. 0 = non-preserved collagen. 1 = preserved collagen. (**a**) Distribution in the human sample. (**b**) Distribution in the animal sample.

For both animals and humans, the countries with the lowest amount of preserved remains are those lying along the southern margin of the Mediterranean Basin (Fig. 4), suggesting that the latitude at which samples were buried influenced their preservation. However, Greece and Spain show the highest amount of well-preserved animal and human samples, respectively. Therefore, collagen is still well preserved and reliable for isotopic analysis even when human and animal remains are buried in (semi-)arid conditions^67–70^. However, it is possible that some of these studies only publish part of the generated dataset and therefore exclude data that do not meet the quality criteria. This may bias the meta-analysis presented here and calls for urgency in publishing both accepted and rejected measurements (according to the quality criteria) in the future. Additionally, we should notice that these findings also follow the geographical data distribution, with the majority of preserved remains coming from those countries with the highest number of samples analysed (Spain and Greece). In contrast, countries with the lowest number of samples studied (e.g. Algeria) have the lowest amount of preserved data.

The analyses shown here are limited to carbon and nitrogen values, given their abundance in the dataset and the absence of such corroborated quality criteria for other measurements (excluding sulphur).

We would recommend users of the dataset cite the original publications as well as this dataset when using data from MAIA.

Researchers are welcome to integrate this dataset with other existing datasets. This is facilitated by the presence of both chronological and geographic coordinates, as well as references to the original publications, that will allow comparison with data from other public repositories.

## Data Availability

No custom code was used.
